# Antioxidant Activity and Acetylcholinesterase Inhibition of Grape Skin Anthocyanin (GSA)

**DOI:** 10.3390/molecules19079403

**Published:** 2014-07-03

**Authors:** Mehnaz Pervin, Md. Abul Hasnat, Yoon Mi Lee, Da Hye Kim, Jeong Eun Jo, Beong Ou Lim

**Affiliations:** College of Biomedical & Health Science, Department of Applied Biochemistry, Konkuk University, Chungju 380-701, Korea; E-Mails: mehnaz.pervin@gmail.com (M.P.); noman33ju@gmail.com (M.A.H.); leeyoonmi83@gmail.com (Y.M.L.); junfeel@kku.ac.kr (D.H.K.); whwjddms90@kku.ac.kr (J.E.J.)

**Keywords:** anthocyanin, antioxidant, free radicals, DNA damage, mitochondria, antioxidant enzyme activity

## Abstract

We aimed to investigate the antioxidant and acetylcholinesterase inhibitory activities of the anthocyanin rich extract of grape skin. Grape skin anthocyanin (GSA) neutralized free radicals in different test systems, such as 2,-2'-azinobis-(3-ethylbenzothiazoline-6-sulfonic acid) (ABTS) and 2,2-diphenyl-1-picrylhydrazyl (DPPH) assays, to form complexes with Fe^2+^ preventing 2,2'-azobis(2-amidinopropane) dihydrochloride (AAPH)-induced erythrocyte hemolysis and oxidative DNA damage. Moreover, GSA decreased reactive oxygen species (ROS) generation in isolated mitochondria thus inhibiting 2',-7'-dichlorofluorescin (DCFH) oxidation. In an *in vivo* study, female BALB/c mice were administered GSA, at 12.5, 25, and 50 mg per kg per day orally for 30 consecutive days. Herein, we demonstrate that GSA administration significantly elevated the level of antioxidant enzymes in mice sera, livers, and brains. Furthermore, GSA inhibited acetylcholinesterase (AChE) in the *in vitro* assay with an IC_50_ value of 363.61 µg/mL. Therefore, GSA could be an excellent source of antioxidants and its inhibition of cholinesterase is of interest with regard to neurodegenerative disorders such as Alzheimer’s disease.

## 1. Introduction

Free radicals or reactive oxygen species (ROS) play many important roles in physiological and pathological processes. Oxidative stress is a biological phenomenon that results from a biochemical imbalance between the formation and clearance/buffering of free radicals [1]. Mitochondria are the major source of cellular ROS. The accumulation of ROS induces oxidative damage of mitochondrial DNA (mtDNA), proteins, and lipids, and has been shown to contribute to the decline in physiological function of cells resulting in a variety of diseases and accelerated aging [2].

Enzymatic systems in cells and body fluids regulate the level of ROS, which otherwise might generate a cascade of products and lead to assailing oxidants. The main classes of antioxidant enzymes in our antioxidant defense system are the superoxide dismutases (SOD), catalases (CAT), and glutathione peroxidases (GPx) [3]. Under oxidative stress conditions, antioxidant enzymes modulate the activities of these ROS and play a role in vascular function [4].

Acetylcholinesterase (AChE) is a hydrolase that plays a key role in cholinergic transmission by catalyzing the rapid hydrolysis of the neurotransmitter acetylcholine (ACh) [5]. Natural products might slow the progression of Alzheimer’s disease (AD) by simultaneously protecting neurons from oxidative stress and acting as cholinesterase inhibitors [6].

Antioxidant supplementation is one plausible strategy to maintain redox homeostasis by directly quenching excessive ROS and protecting or reinforcing endogenous antioxidative defense systems against oxidative stress [7]. At present, many antioxidants are synthetically manufactured. They may possess some side effects and toxic properties to human health [8]. Therefore, studies of antioxidant substances in foods and natural products have gained increasing interest.

Anthocyanins are flavonoids, a class of secondary plant metabolites, with phenolic groups in their chemical structure that are responsible for the pigmentation in several different fruits. In grapes, they are found almost exclusively in the skins, with only a limited number of varieties showing these compounds in the pomace [9]. Recently, a great number of researchers have identified and characterized various anthocyanins found in grape skin with Liquid chromatography–mass spectrometry (LC/MS). The main *Vitis vinifera* grape anthocyanins, cyanidin, malvidin, delphinidin, petunidin, and peonidin, are present as monoglucoside, acetylmonoglucoside, and *p*-coumaroylmonoglucoside derivatives [10]. The most common anthocyanin in *V. vinifera* is malvidin-3-*O*-glucoside [11]. Choi *et al*. [12] showed that anthocyanins are primarily responsible for the antioxidant activity of this grape variety, which was also reported in other grape varieties. Anthocyanins are used in the treatment of coronary heart disease and are administered as dietary supplements for the prevention and treatment of metabolic disorders, in particular, obesity, diabetes, and osteoarthritis [13]. Anthocyanins also possess anticancer and antineurodegenerative properties [14,15,16]. It has been shown that anthocyanins have beneficial effects on memory and cognition, suggesting a clear neuroprotective role [17,18].

Therefore, the primary objective of our research was to investigate the antioxidant effects of grape skin anthocyanins using various *in vitro* and *in vivo* methods. In this study, physiologically relevant antioxidant activities of GSA, including erythrocyte membrane protection, DNA protection, and mitochondria protection were investigated for the first time. Moreover, we studied the *in vivo* antioxidant activity of GSA in mice serum, liver, and brain. Another major objective was to determine potential *in vitro* anticholinesterase activity of GSA.

## 2. Results and Discussion

### 2.1. Radical Scavenging Activity

GSA’s free radical scavenging activity was evaluated on different free radical species: DPPH and ABTS. GSA was able to reduce the stable radical DPPH to the yellow-colored diphenyl picryl hydrazine. GSA exhibited a significant concentration-dependent inhibition of DPPH activity, with a 50% inhibition (IC_50_) at a concentration of 95.54 µg/mL. The results are presented in Table 1. The IC_50_ value of vitamin C was 71.50 µg/mL. These results indicate that GSA might act as an electron or hydrogen donator to scavenge DPPH^•^ radicals.

The ABTS radical scavenging assay is shown in Table 1. GSA showed scavenging activity in a concentration-dependent manner. The concentration for 50% inhibition of GSA and vitamin C were found to be 62.74 and 20.32 µg/mL, respectively. These results indicate that GSA has strong scavenging power for ABTS radicals and should be explored as a potential antioxidant. Previous studies have confirmed the free radical scavenging activity of red grape pomace seeds and skin extracts [19].

**Table 1:** DPPH, ABTS radical scavenging and metal chelating activities of GSA.

Extract	DPPH Radical		ABTS Radical		Metal Chelating	
	Scavenging Activity (%)	IC_50_ Value µ g/mL	Scavenging Activity (%)	IC_50_ Value µ g/mL	Scavenging Activity (%)	IC_50_ Value µ g/mL
GSA	95.54 ± 0.43 ^a^	95.14 ± 1.13 ^a^	97.67 ± 1.009 ^a^	62.74 ± 0.43 ^a^	56.26 ± 1.67 ^a^	180.49 ± 19.40 ^a^
Positive control	97.75 ± 0.28 ^b^ (Vitamin C)	71.50 ± 1.05 ^b^	99.78 ± 0.34 ^b^ (Vitamin C)	20.32 ± 0.20 ^b^	89.82 ± 2.69 ^b^ (EDTA 100 µM)	7.089 ± 0.78 ^b^

All data are expressed as mean ± SD (*n* = 3). Different letters in each column denote statistically significant difference compare to the positive control group at *p* < 0.05. Scavenging activity (%) was determined at 500 µg/mL.

### 2.2. Fe^2+^ Chelation

Chelation is an important parameter because iron is required for oxygen transport, respiration, and the activities of many enzymes. However, iron is an extremely reactive metal and can catalyze oxidative changes in lipid, proteins, and other cellular components. Fe^2+^ ion can trigger a Fenton reaction when it encounters H_2_O_2_, and the product of this reaction (hydroxyl radical) can cause great oxidative damage [20]. Therefore, ferrous ion-chelating activity is considered an important indicator in any oxidative stress involving ferrous ion. Fe^2+^ ion is the most powerful pro-oxidant among the various species of metal ions [21]. Ferrozine can quantitatively form complexes with Fe^2+^. However, in the presence of chelating agents, the complex formation is disrupted, resulting in a decrease in the red color of the complex. Measurement of color reduction therefore allows for estimation of the metal chelating activity of the co-existing chelator. The capacity of GSA to chelate Fe^2+^ is shown in Table 1. We found that GSA could chelate Fe^2+^ efficiently and therefore reduce the production of free radicals. The IC_50_ value for GSA’s chelating abilities was 180.49 µg/mL. EDTA was used as reference in this assay and its IC_50_ value for Fe^2+^-chelation was 7.08 µg/mL.

### 2.3. Oxidative Hemolysis Inhibition Assay

**Figure 1:**
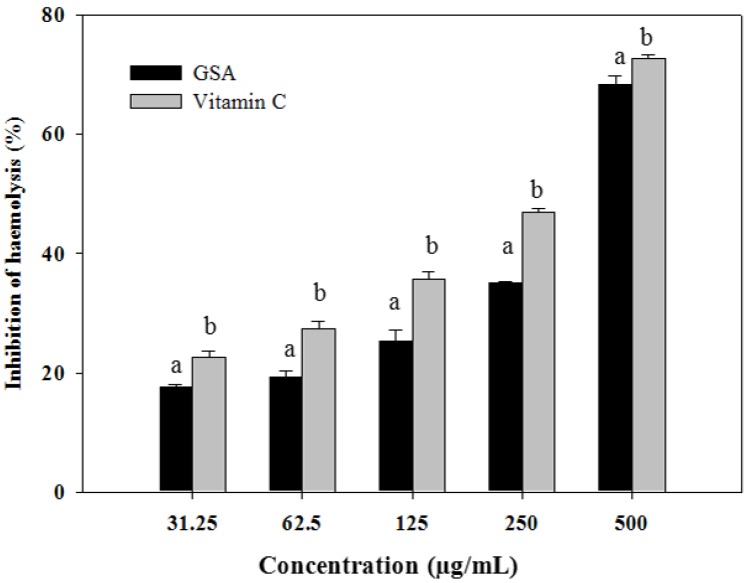
Anti- haemolytic activity of GSA on APPH-induced erythrocyte haemolysis *in vitro*. Data are expressed as mean ± SD (*n* = 3). Columns with different letters are significantly different at *p* < 0.05 level.

The oxidative hemolysis inhibition assay system is based on the property of erythrocytes that renders them susceptible to oxidative damage and utilizes the biologically relevant radical source, AAPH-derived peroxyl radicals, to attack the erythrocyte membrane and cause erythrocyte hemolysis [22]. The rate of cell lysis can be regarded as an *in vitro* marker of oxidative damage. As shown in Figure 1, inhibition rates of erythrocyte hemolysis were 17.63%, 19.36%, 25.37%, 35.12%, 68.35%, and 68.35% for GSA, and 22.54%, 27.47%, 35.70%, 46.85%, and 72.89% for vitamin C at the tested concentrations of 31.25, 62.5, 125, 250, and 500 µg/mL, respectively. Our results are in agreement with other studies showing that polyphenols are able to protect erythrocytes from oxidative stress or increase their resistance to damage caused by oxidants [23,24].

### 2.4. Oxidative DNA Damage Prevention

**Figure 2:**
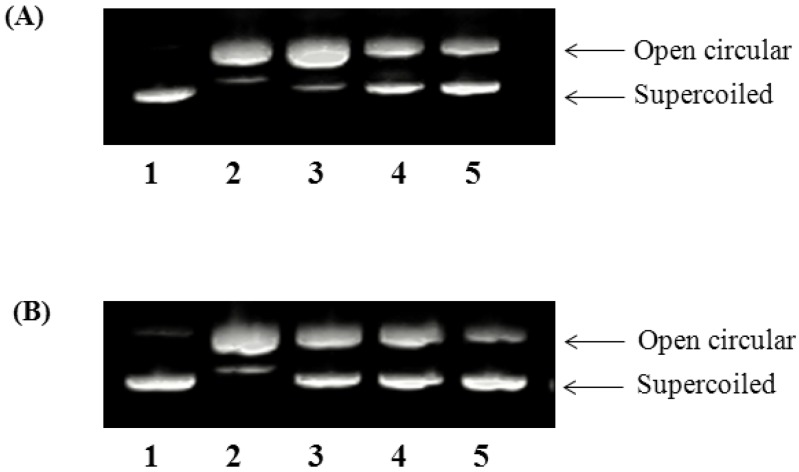
Protective effect of GSA on hydroxyl radical-mediated pBR322 DNA strand breaks. (**A**) GSA (**B**) Vitamin C. Lane 1: normal DNA control; lane 2: FeSO_4_ + H_2_O_2_ (DNA damage control); lane: 3–5: FeSO_4_ + H_2_O_2_ + DNA in the presence of GSA (125, 250 and 500 µg/mL, respectively).

ROS, such as superoxide anion (O_2_^−^), hydrogen peroxide (H_2_O_2_), and hydroxyl radical (^•^OH) can cause damage to biological macromolecules leading to lipid peroxidation, protein oxidation, and DNA base modification and strand breaks [21]. Permanent modification of DNA as a result of oxidative damage is the first step in several pathological and physiological conditions such as cancer and aging, respectively. The inhibition of H_2_O_2_-induced DNA strand breakage by GSA was assessed by measuring the conversion of the supercoiled pBR322 plasmid DNA to open circular and linear forms by gel electrophoresis. Because hydroxyl radical (^•^OH) modifies and destroys DNA in a nonspecific manner, protection capacity against ^•^OH-induced oxidation of DNA was also measured to evaluate an antioxidant. Figure 2, shows the inhibitory effect of GSA on pBR322 plasmid DNA cleavage caused by H_2_O_2_. Conversion of the supercoiled form of this plasmid DNA to the open-circular and further linear forms has been used as an index of DNA damage. The plasmid DNA was mainly in the supercoiled form in the absence of Fe^2+^ and H_2_O_2_ (lane 1, control). After the addition of Fe^2+^ and H_2_O_2_, the quantity of supercoiled DNA decreased due to conversion into the relaxed circular and linear forms. However, further fragmentation of linear form decreased in the presence of GSA (125–500 µg/mL). Both GSA and vitamin C were concentration–dependent for preventing DNA damage. The observed scission-inhibition could be due to the scavenging of hydroxyl radicals by GSA. Devasagayam *et al*. [25] studied the ability of natural antioxidants, such as carotenoids and flavonoids to protect the pBR322 plasmid DNA against ROS. In a previous study Noroozi *et al*. [26] reported that flavonoids and vitamin C were effective against hydrogen peroxide initiated oxidative DNA damage to human lymphocytes.

### 2.5. Evaluation of Antioxidation of GSA Using a Mitochondria-Based Assay

Approximately 90% of cellular ROS are produced in the mitochondria [27]. ROS levels are thought to increase with age owing to the accumulation of damaged mitochondria in a self-perpetuating cycle. ROS-induced impairment of mitochondria results in increased ROS production, which in turn leads to further mitochondrial damage [28]. Measurement of ROS in living organisms has been a significant analytical challenge. Most ROS are highly reactive and short lived and therefore are difficult to detect in complex biological matrices. Additionally, ROS are often produced and/or neutralized in subcellular compartments, which requires detection methods specific to subcellular localization [29].

**Figure 3:**
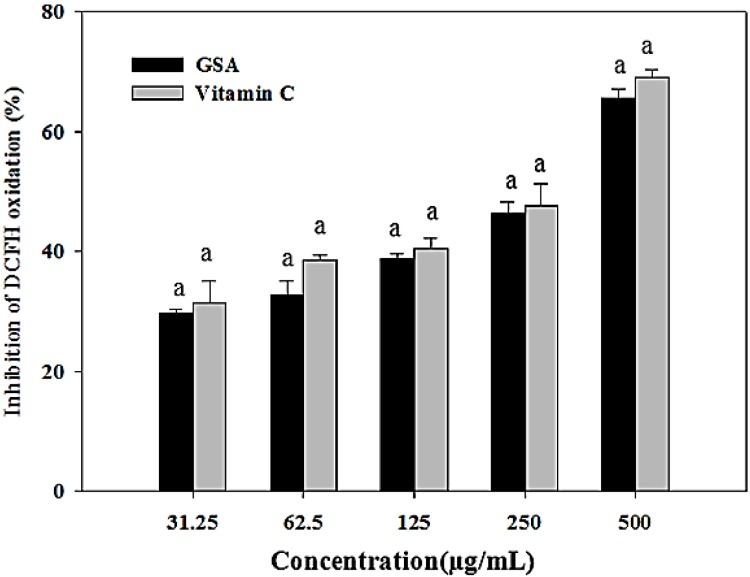
Protective efffect of GSA against oxidative damage on isolated mouse liver mitochondria. ROS generation was assayed as inhibition of DCFH oxidation. Values represent the mean ± SD (*n* = 3). Columns with different letters are significantly different at *p* < 0.05 level.

A physiologically relevant mitochondria-based assay was used to assess the antioxidant capability of GSA against oxidative stress in mitochondria. Ascorbic acid was used as the reference antioxidant. GSA could inhibit DCFH oxidation by scavenging ROS, thus resulting in decreased fluorescence intensity. As illustrated in Figure 3, the tested sample and ascorbic acid standard exhibited strong antioxidant capacity in a concentration-dependent manner. Inhibition of DCFH oxidation was 29.59%, 32.78%, 38.73%, 46.22%, and 65.62% for GSA, and 31.40%, 38.46%, 40.40%, 47.61%, and 68.95% for vitamin C at the tested concentrations of 31.25, 62.5, 125, 250, and 500 µg/mL, respectively (Figure 3). GSA did not show any significant difference compared to vitamin C (*p* < 0.05). The method for monitoring H_2_O_2_ generation in isolated mitochondria by DCFH-DA chemical probe was first introduced in 1983 [30]. It is known that once DCFH-DA enters the cell, the acetyl moiety is cleaved by intracellular esterases; subsequent oxidation by ROS, particularly H_2_O_2_ and hydroxyl radical, yields the fluorescent product, DCF. The principle of this method is that antioxidants can scavenge ROS generated in mitochondria, thus inhibiting DCFH oxidation.

### 2.6. Antioxidant Activities in Vivo

**Figure 4:**
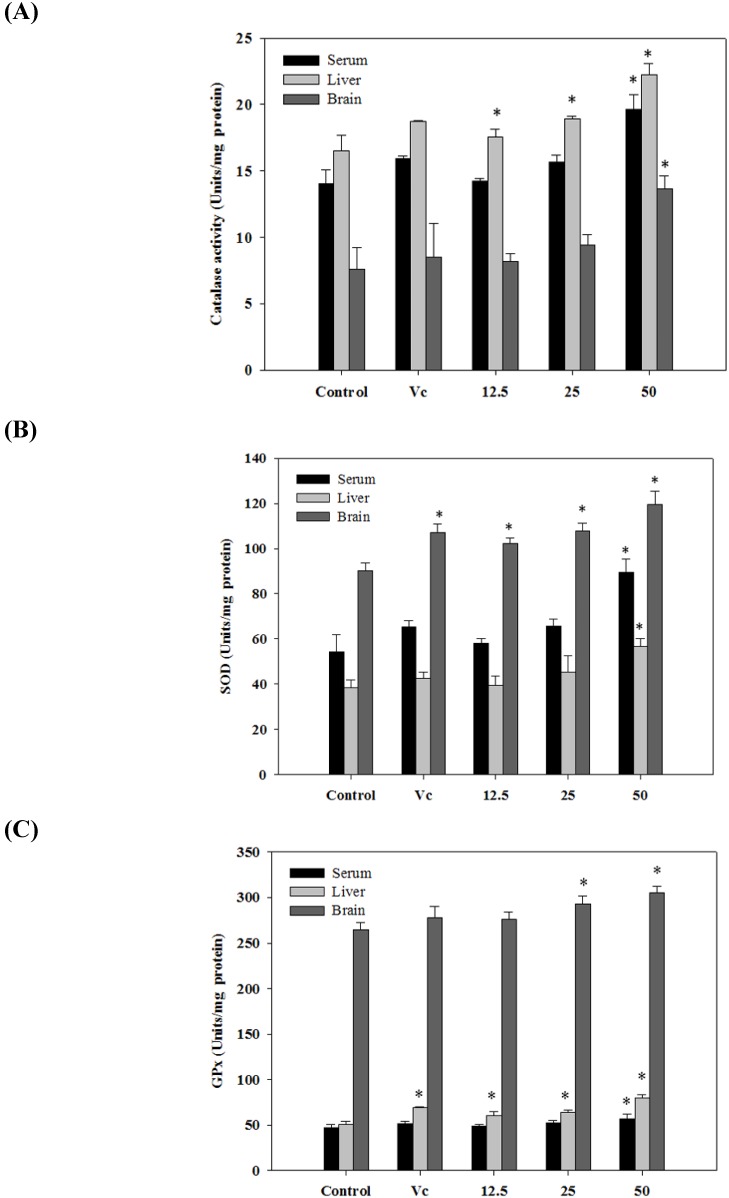
Effects of GSA on level of catalase (CAT) (**A**) SOD (**B**) and GPx (**C**) in serum, liver and brain of mice. Values are the mean ± SD (*n* = 5); * *p* < 0.05 compared with normal control group. All activities were expressed as unit per milligram of protein (U/mg protein).

The cooperative defense systems that protect the body from free radical damage include antioxidant nutrients and enzymes. As shown in Figure 4, after administration of GSA (50 mg/kg) SOD, CAT, and GPx activities were noticeably increased in mice serum, liver, and brain than those in the control group (*p* < 0.05). In most of the cases, compared with the control group, levels of antioxidant enzymes were not significantly elevated for the GSA extract at 12.5 and 25 mg/kg (*p* < 0.05). Administration of ascorbic acid (50 mg/kg) also showed significant increase in SOD, CAT, and GPx levels. These data suggest that GSA has significant effects on the levels of antioxidant enzymes in mice.

Regarding the *in vivo* study, evidence has shown that ethanolic extract of white button mushroom (*Agaricus bisporus*)-fed mice led to a significantly higher level of antioxidant enzymes (SOD, GsH-Px, and CAT) in mice serum, liver, and heart [31]. Grape skin anthocyanin activates the antioxidant enzymes SOD, CAT, and GPx in H_2_O_2_ treated retinal cells [32]. Puiggros *et al*. [33] provided evidence that grape seed procyanidin extract increased the Cu/Zn-SOD activity in rats and Fao cell line hepatocytes. In *in vivo* assay, numerous factors such as bioavailability, digestibility, and metabolism of the compound may influence biological potentials. Previous studies indicated that anthocyanin can rapidly reach the plasma after oral administration. [34,35]. Two previous works reported the capacity of dietary anthocyanins from grapes and berries to reach the brain [36,37]. Moreover, the results of a previous clinical study suggested that antioxidative anthocyanins are obviously absorbed from grape juice and wine [38].

### 2.7. In Vitro Cholinesterase Inhibition

**Figure 5:**
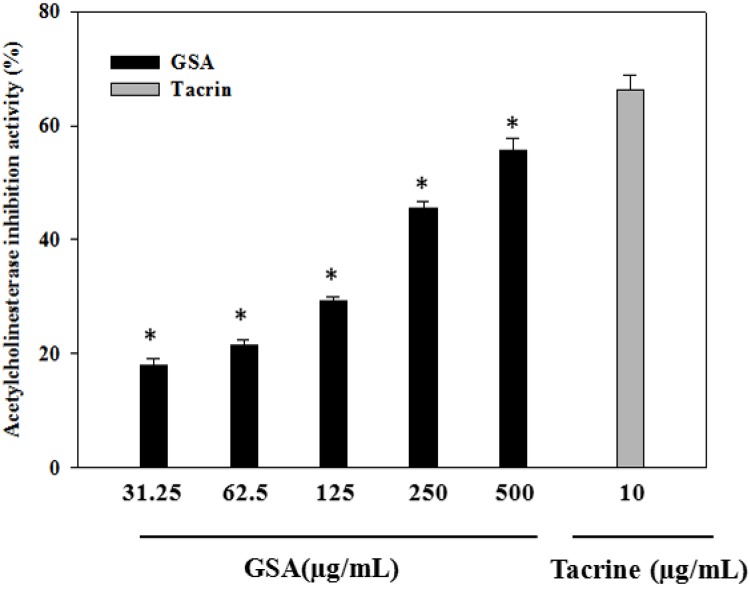
Acetylcholinesterase inhibitory activity of GSA. Values represent the mean ± SD (*n* = 3). * *p* <0.05, compare to the positive control group.

Acetylcholinesterase (AChE) is a hydrolase that plays a key role in cholinergic transmission by catalyzing the rapid hydrolysis of the neurotransmitter acetylcholine (ACh) [5]. The use of acetylcholinesterase inhibitors elicits numerous responses, which mediate the symptoms of Alzheimer’s disease [39]. When studied for its possible inhibitory effect in the *in vitro* assay, GSA showed AChE inhibitory activity in a dose-dependent manner. As illustrated in Figure 5, at tested concentrations of GSA (31.25–500 µg/mL), acetylcholinesterase inhibitory activities were 17.94%, 21.47%, 29.16%, 45.57%, and 55.58%, respectively (IC_50_ = 363.61 µg/mL). Tacrine was used as a reference inhibitor and was more active than GSA (*p* < 0.05). At 10 µM tacrine showed a 66.30% inhibition of AChE. Several studies recently supported that different plant extracts and active compounds, including anthocyanins (pelargonidin, delphinidin and cyanidin), terpenoids, also have anticholinesterase activity [40,41,42]. The leaves of pomegranate and grapes exhibited considerable acetylcholinesterase inhibitory activity [43,44]. Several studies showed that grape and blueberry anthocyanin have clear neuroprotective roles [16,18,45].

## 3. Experimental Section

### 3.1. Samples and Chemicals

Anthocyanin rich grape skin extract was manufactured by Kitolife (Pyeong-Teak, Korea). The anthocyanin content of grape skin (*Vitis vinifera* L.) extract was standardized at 80% (w/w) by high-performance liquid chromatography (HPLC) system. These products were manufactured according to a previously described method [32]. In brief, grap skin (*V. vinifera* L. cv. Aglianico) were collected and extracted in methanol (0.75% HCl) solution for five days at room temperature. Anthocyanins extracted from skins of grape contain the following four major compounds: malvidin 3-*O*-glucoside, petunidin 3-*O*-glucoside, delphinidin 3-*O*-glucoside, and cyaniding 3-*O*-rutinoside [13,32,46].

2,2-Diphenyl-1-picrylhydrazyl (DPPH), 2,2'-azinobis-(3-ethylbenzothiazoline-6-sulphonic acid) diammonium salt (ABTS), gallic acid, sodium nitrite, Folin–Ciocalteu reagent (FC reagent), butylated hydroxyluene (BHT), ascorbic acid (AA), α-tocopherol, potassium persulphate, ferrous chloride, ammonium thiocyanate, ethylene-di-amino-tetraacetic acid (EDTA), linoleic acid, anhydrous sodium phosphate (dibasic), anhydrous sodium phosphate (monobasic), 5,5'-dimethyl-pyrroline-1-oxide (DMPO), pyrogallol and ferrous sulphate (FeSO_4_), ethylene-bis-(oxyethylenenitrilo)-tetraacetic acid (EGTA), HEPES, glutamate, succinate, and 2',7'-dichlorofluorescin diacetate (DCFH-DA) were purchased from Sigma-Aldrich (St. Louis, MO, USA). Sodium hydroxide and ferric chloride were obtained from Wako Pure Chemical Industries Ltd. (Osaka, Japan). The catalase assay kit and SOD assay kit were purchased from Cayman Chemical Company (Ann Arbor, MI, USA). The pBR322 DNA and 6× DNA loading dye were purchased from Fermentas Inc. (Cromwell Park, Glen Burnie, USA). All other reagents were of analytical grade.

### 3.2. 2,2-Diphenyl-1-picrylhydrazyl (DPPH) Radical Scavenging Activities

Free radical scavenging activity on DPPH by GSA was assessed by a previously described colorimetric method [20]. In brief, an 80 μL aliquot of sample solution at different concentrations (31.25–500 µg/mL) was mixed with 80 μL DPPH solution (0.3 mM in methanol). The reaction mixture was incubated for 30 min in the dark at room temperature. The absorbance of the resulting solution was measured at 517 nm with a spectrophotometer (Sunrise-Basic Tecan, Salzburg, Austria). Controls were prepared in a similar manner using the corresponding extraction solvent in place of the sample solution. The radical scavenging capacity of the tested samples was measured using the following equation:

Scavenging activity (%) = (1 − Absorbance of sample/Absorbance of control) × 100

### 3.3. 2,2'-Azinobis-(3-ethylbenzothiazoline-6-sulfonic acid) (ABTS) Radical-Scavenging Activity

Free radical scavenging activity on ABTS by GSA was determined using the method described by He *et al*. [47] with slight modifications. The ABTS^•+^ radical was generated by the reaction of 7 mM 2,2'-azinobis-(3-ethylbenzothiazoline-6-sulphonic acid) diammonium salt (ABTS, 5 mL) with 2.45 mM of potassium persulphate (88 µL). The mixture was left to stand for 12–16 h in the dark at room temperature. Absorbance of the reactant was later adjusted to 0.70 ± 0.02, at room temperature, at a wavelength of 734 nm. Different concentrations of tested extract were mixed with 0.7 mL of ABTS^•+^ solution and the mixture was shaken for 5 min. The reduction of the ABTS^•+^ radical was determined by reading the absorbance at 734 nm using a UV spectrophotometer (Pharmaspec UV-1700, Shimadzu, Kyoto, Japan). The controls contained the extraction solvent instead of the test sample. The scavenging activity of ABTS free radical was calculated as:

Scavenging activity (%) = (1 − Absorbance of sample/Absorbance of control) × 100

### 3.4. Fe^2+^ Chelation Assay

The ferrous ion-chelating activity of GSA was estimated in accordance with the method described by Cheng *et al*. [20]. GSA (31.25–500 µg/mL). Each sample was incubated with 50 µL of 2 mM FeCl_2_ for 5 min. The reaction was initiated by adding 200 µL of 5 mM ferrozine. After incubation for 5 min at room temperature, the absorbance of the mixture was measured at 562 nm against the blank, which was performed in the same way using FeCl_2_ and water. EDTA (3.12–100 µg/mL) served as the positive control, and a sample without the sample or EDTA served as the negative control. The Fe^2+^-chelating activity was calculated using the equation below:

Chelating activity (%) = (1 − Absorbance of sample/Absorbance of control) × 100

### 3.5. Oxidative Hemolysis Inhibition Assay

Anti-hemolytic activity was assayed according to the method described by Carvalho *et al*. [23]. Blood was collected from female BALB/c mice (weighing 20 ± 2 g). RBCs were separated from plasma by centrifugation at 1500 *g* for 20 min. The crude RBC was then washed five times with five volumes of phosphate-buffered saline (PBS, pH 7.4). The RBC was suspended in four volumes of PBS solution for hemolysis assay. Two mL of RBC suspension were mixed with 2 mL of PBS solution containing GSA (31.25–500 µg/mL). The erythrocyte suspension was agitated gently while being incubated with APPH (final concentration, 50 mM) at 37 °C for 3 h. After incubation, 8 mL of PBS solution was added to the reaction mixture, followed by centrifugation at 1,000 *g* for 10 min. The absorbance of the supernatant was recorded at 540 nm in a spectrophotometer. Percentage inhibition was calculated by the following equation:

% Inhibition = (1 − Absorbance of sample/Absorbance of control) × 100

### 3.6. Assay for Effects of GSA on DNA oxidative damage

The protective effect of GSA on DNA strand breaks induced by hydroxyl radicals was measured by the conversion of pBR322 DNA to an open circular form according to the method described by Cheng *et al*. [20] with some minor modifications. Briefly, 1 μL of plasmid pBR322 DNA (0.5 μg/μL) was treated with 3 μL of FeSO_4_ (0.08 mM), 4 μL of 30% H_2_O_2_ (v/v), 3 μL distilled water, and 2 μL of the tested sample at different concentrations (125–500 µg/mL). The mixture was then incubated in a water bath at 37 °C for 1 h. Then 2 μL of DNA loading dye (6×) was added to the mixture. The DNA samples were resolved on a 0.8% (w/v) agarose gel using ethidium bromide staining. Gels were scanned on a gel documentation system (Nextep, Seoul, Korea) and bands were quantified using NEXTEP analysis software. To avoid photolysis of samples, experiments were conducted in the dark.

### 3.7. Mitochondria-Based Assay

#### 3.7.1. Isolation of Mitochondria from Liver

Mitochondria were isolated from the livers of mice according to the methods described by He *et al*. [47], with some modifications. Briefly, livers were rinsed using cold homogenization media, and subsequently homogenized in the homogenization buffer A (1:4, w/v; sucrose 0.32 mol/L, EDTA 1 mmol/L, Tris–HCl 10 mmol/L, and bovine serum albumin (BSA) 65 mmol/L, pH 7.4). This homogenate was centrifuged for at 45 *g* for 10 min, and the unbroken tissue, cells, and nuclei were discarded. The supernatant obtained was centrifuged at 15,000 *g* for 10 min, and the pellet was collected and resuspended in the homogenization buffer A. This procedure was repeated until a single pellet was obtained. The pelleted mitochondria were resuspended in 30 mL of buffer B (KCl 137 mmol/L, HEPES 10 mmol/L, MgCl2 2.5 mmol/L, and EDTA 0.5 mmol/L, pH 7.2) and stored at −20 °C until use. The concentration of mitochondrial protein was determined using the Bradford protein assay with BSA as a standard.

#### 3.7.2. Mitochondrial Reactive Oxygen Species (ROS) Measurements

ROS production in mitochondria was measured using 2',7'-dichlorofluorescin diacetate (DCFH-DA), a H_2_O_2_-sensitive fluorescent probe, as previously described by He *et al*. [47], with modifications. Briefly, 40 μL of appropriate dilutions of extract was added into a mixture containing 30 μL glutamate (40 mmol/L), 30 μL succinate (40 mmol/L) and 165 μL H_2_O_2_ buffer in a 96-well plate, and followed by 75 μL DCFH-DA (52 μmol/L). Then, 60 μL (1.5 mg/mL) mitochondrial suspension was added to initiate the reaction, which was incubated at 37 °C for 10 min. The change in fluorescence of the reaction mixture was recorded at 485 nm excitation and 530 nm emission in a spectrophotometer. Inhibition of DCFH oxidation was calculated by the following equation:

% Inhibition = (1 − Absorbance of sample/Absorbance of control) × 100

### 3.8. In Vivo Antioxidant Activity

#### 3.8.1. Animals and Experimental Design

*In vivo* antioxidant activity was assayed according to the methods described by Liu *et al*. [31] with some modifications. Female BALB/c mice (weighing 20 ± 2 g, 8 weeks old) were purchased from Orient Bio Inc. (Seongnam, Gyeonggi, Korea). Animals were acclimatized under controlled conditions for 1 week before experimental feeding. Mice were housed in specific pathogen-free conditions in an animal room at Konkuk University, maintained on a 12-h light/dark cycle, and provided food and water *ad libitum*. All animal procedures were carried out according to a protocol approved by the Institutional Animal Care and Use Committee of the Konkuk University. After one week of adaptation, the mice were randomly divided into five groups of 5 animals each: Group 1 (control) received vehicle (water), Group 2 received vitamin C (positive control), Group 3, Group 4, and Group 5 received GSA at 12.5, 25, and 50 mg/kg body weight, respectively, by gavage for 30 consecutive days. 

#### 3.8.2. Biochemical Assay

Twenty-four hours after the last drug administration, mice were sacrificed and blood samples were collected. The blood samples were then centrifuged at 10,000 *g* at 4 °C for 10 min to obtain blood serum, which was then stored at −80 °C for further analysis. The liver and brain were removed immediately, washed and homogenized in ice-cold physiological saline to prepare a 10% (w/v) homogenate. Then, the homogenate was centrifuged at 1,000 *g* at 4 °C for 10 min to remove cellular debris, and the supernatant was collected for analysis. 

Antioxidant enzymatic activities were determined using SOD, CAT, and GPx assay kits (Cayman Ann Arbor, MI, USA) following the manufacturer’s instructions. One unit of SOD is defined as the amount of enzyme needed to exhibit 50% dismutation of the superoxide radical. Detection of catalase activity was based on the reaction of the enzyme with methanol in the presence of an optimal concentration of H_2_O_2_. The formaldehyde produced was measured spectrophotometrically with 4-amino-3-hydrazino-5-mercapto-1,2,4-triazole as the chromogen. Catalase activity was expressed as µmol of formaldehyde per min per g of protein from homogenates. GPx activity was measured on the basis of the reaction of GSH and 5,5'-dithiobis-(2-nitrobenzoic acid). The protein contents in the supernatants obtained from the liver and brain were determined by the Bradford Protein assay kit. All the above treatments were performed at 4 °C.

### 3.9. In Vitro Anticholinesterase Inhibition Assay

The AChE inhibition assay was carried out in a multi-well plate using a modified method, as described by Ellman *et al*. [48]. Electric eel acetylcholinesterase was used, while acetyl thiocholine iodide (ATCI) was used as the substrate of the reaction. 5,5-dithiobis(2-nitrobenzioc) acid (DTNB) was used for measurement of AChE activity. Briefly, 150 μL of 0.1 M sodium phosphate buffer (pH 8.0), 10 μL test compound solution, and 20 μL of enzyme solution (0.09 units/mL) were mixed and incubated for 15 min at 25 °C. 10 μL of DTNB (10 mM) was then added and reaction was initiated by the addition of substrate (10 μL of ATCI, 14 mM solution). The hydrolysis of the ATCI can be measured by the formation of the product, 5-thio-2-nitrobenzoate, a colored anion formed by the reaction of DTNB and thiocholine, which is released by enzyme hydrolysis. Absorbance was measured at 412 nm (Shimadzu, 1200, Japan) after 10 min. Tacrine, a standard AChE inhibitor, was used as positive control. The percent of acetylcholinesterase inhibition was calculated as following:

% Inhibition = 100 − [Absorbance of the test compound/Absorbance of the control] × 100

### 3.10. Statistical Analysis

Data are expressed as mean ± standard deviation (SD). All analysis was carried out in at least three replicates for each sample. Results were analyzed statistically using SPSS 15.0, Sigma plot 10.0, and GraphPad Prism 5 software (San Diego, CA, USA). A value of *p* < 0.05 was considered statistically significant.

## 4. Conclusions

GSA possesses strong antioxidant activity as demonstrated by biologically relevant assays, such as the oxidative DNA damage prevention assay, hemolysis inhibition assay, liver mitochondria oxidative damage prevention assay. Administration of GSA could significantly enhance the activities of antioxidant enzymes (SOD, CAT, and GPx) in mice sera, liver, and brain. Moreover, the results suggest that GSA can inhibit cholinesterase activities. Altogether, our results show that GSA has great value for preventing oxidative stress-related disease and can be a prominent source of anticholinesterase activity.
